# Computed tomography image quality in patients with primary hepatocellular carcinoma: intraindividual comparison of contrast agent concentrations

**DOI:** 10.3389/fmed.2024.1460505

**Published:** 2024-10-16

**Authors:** Fei Peng, Chaotian Luo, Xiaojing Ning, Fangyan Xiao, Kaiming Guan, Cheng Tang, Fuling Huang, Junli Liang, Peng Peng

**Affiliations:** Department of Radiology, The First Affiliated Hospital of Guangxi Medical University, Nanning, China

**Keywords:** contrast agent, iodine concentration, hepatocellular carcinoma, comparative study, computed tomography

## Abstract

**Objective:**

This study aimed to assess the impact of the different concentrations of iodine contrast agents used on the quality of computed tomography (CT) images obtained intraindividually in hepatocellular carcinoma patients.

**Methods:**

In this retrospective study, data from a cohort of 29 patients diagnosed with primary hepatocellular carcinoma who had undergone two preoperative CT-enhanced examinations within a 3-month timeframe were analyzed. Each patient was randomly assigned to receive either a low-concentration contrast agent (300 mg I/mL iohexol) or a high-concentration contrast agent (350 mg I/mL iohexol) for the first scan and the alternative contrast agent for the second scan. CT images of different liver regions of each patient were compared between low-and high-concentration scans using their before-and-after control design. Subjective image quality scores for portal vein images were also assessed.

**Results:**

The findings of this study indicate that patients in the high-concentration group presented significantly elevated CT values across various anatomical regions, including the liver parenchyma, abdominal aorta, and hepatic portal vein, compared to those in the low-concentration group (*p* < 0.05). Moreover, the high-concentration group demonstrated superior subjective image ratings (*p* < 0.05). Nevertheless, there was no statistically significant difference in the CT values observed in liver cancer parenchyma scans at different phases between the two groups (*p* > 0.05).

**Conclusion:**

In summary, using a high-concentration iodine contrast agent is efficient in enhancing the visual clarity of the liver parenchyma, the aorta, and the portal vein in individuals diagnosed with primary hepatocellular carcinoma.

## 1 Introduction

Hepatocellular carcinoma (HCC) is the sixth most prevalent neoplastic disorder worldwide and the third leading cause of cancer-related death (1). Early diagnosis of HCC is crucial for improving patient outcomes. With advances in medical imaging technology, dynamic contrast-enhanced computed tomography (CT) is regarded as one of the best non-invasive diagnostic imaging techniques for HCC (2). The quality of CT images is pivotal for tumor detection, staging, and the formulation of treatment plans, and the use of contrast agents is a key element in enhancing image quality.

Several studies, both domestic and international, have demonstrated that the concentration of contrast agent used significantly affects the enhancement of the hepatic vasculature and parenchyma. High-concentration contrast agents offer superior vascular enhancement, thereby improving the contrast between tumors and normal liver tissues. However, these agents typically have relatively high viscosities, which may restrict their iodine delivery rates (IDRs) (3–5). Conversely, low-concentration contrast agents, while generally less viscous, may not provide adequate enhancement to meet clinical demands for image quality (6).

Although previous studies have explored the impact of varying contrast agent concentrations on liver imaging, few have focused specifically on the HCC patient population (7–11). Particularly in clinical practice, selecting the most appropriate contrast agent concentration based on different stages of HCC remains an unresolved issue. Traditional independent sample designs may be susceptible to inter-individual variability. To address this, our study employs an intraindividual control design. This design allows for direct comparison of low-and high-concentration contrast agents within the same patient, effectively controlling for individual differences and enhancing the reliability of the results. Moreover, our research introduces a subjective image quality score, offering a novel perspective to the existing literature, which has not been thoroughly explored in previous studies.

This study aimed to evaluate the CT image quality in HCC patients using different iodine concentrations of contrast media, employing a within-subject before-and-after control design. This approach can more sensitively detect the impact of contrast agent concentration changes on image quality, thereby providing a more optimized protocol for the use of contrast agents in clinical practice.

## 2 Methods

### 2.1 Patient population

This study was conducted in accordance with the principles set forth in the Declaration of Helsinki and was approved by the Medical Ethics Committee of the First Affiliated Hospital of Guangxi Medical University (Approval No: 2024-E159-01); the requirement for informed consent from the participating patients was waived. A retrospective analysis of patients who met specific criteria and were treated at the First Affiliated Hospital of Guangxi Medical University from May 2017 to July 2022 was conducted. The inclusion criteria were as follows: (1) diagnosed with HCC through clinical or pathological confirmation; (2) underwent upper abdominal CT contrast-enhanced examinations with the same scanning parameters and contrast agent injection protocols; (3) underwent imaging follow-up examinations within 3 months using both a low-concentration iodine contrast agent (iohexol) and a high-concentration iodine contrast agent (ioversol); and (4) did not undergo chemotherapy, surgery, interventional therapy, or radiofrequency ablation during the follow-up period. The exclusion criteria were as follows: (1) patients with tumor size change of more than 15% or new metastases during the follow-up period; (2) patients with portal vein thrombosis or vascular anomalies causing abnormal liver perfusion; (3) poor image quality that could affect diagnosis; and (4) incomplete clinical data. This study used a before-and-after controlled study design for each patient; for the first scan, patients were randomly assigned to either the low-concentration group, in which 300 mg I/mL iohexol was used, or the high-concentration group, in which 350 mg I/mL ioversol was used, and for the second scan, they were assigned to the group they had not been in for the first scan. Because each patient was scanned using both low-and high-concentration contrast agents, two sets of data were obtained. The characteristics of the patients are shown in Table 1.

**Table 1:** Demographic and clinical findings related to the study patients (*n* = 29).

Characteristics	Value
Mean age, years	53.52 ± 10.86 (33–77)
Sex	
Male	26
Female	3
Weight, kg	60.25 ± 9.21 (48–80)
Body mass index, kg/m^−2^	22.05 ± 22.05 (17.81–30.11)
Child–Pugh class	
Class A	17
Class B	10
Class C	2
Time interval between serial CT examinations, days	55.19 ± 22.42 (14–90)

Values are presented as the mean ± SD (range).

### 2.2 Image acquisition and contrast medium injection parameters

Prior to the examination, all patients underwent a pre-examination procedure that involved the insertion of a 20-G indwelling needle into the forearm vein. This needle was used before and after the procedure. The patients also received training on how to cooperate by holding their breath. The study utilized a Siemens Somatom Definition Flash dual-source CT scanner (Siemens Healthcare, Forchheim, Germany) for the CT scanning protocol. Prior to the examination, all patients provided informed consent by signing a CT enhancement consent form. The patients were placed in the supine position with their hands elevated toward the sides of the head and neck. The scanning region extended from the upper part of the diaphragm to the lower edge of the liver. The tube voltage was set at 120 kV, and the tube current was automatically determined via the automatic tube current mode on the basis of the patient’s body size. After enhancement, both plain scanning and three-phase scanning were conducted in spiral mode, with a collimator width of 1.2 mm × 32, a pitch of 0.8, a rotation speed of 0.5 s/rotation, an acquisition matrix of 256 × 256, a layer thickness of 5 mm, and a layer spacing of 5 mm.

After the routine scan, a high-pressure injector was employed to administer contrast agents intravenously via the right antecubital vein of the forearm. For the initial scan, the contrast agent used was either iohexol (300 mgI/mL) provided by Beijing Beilu Pharmaceuticals Co., Ltd., or iohexol (350 mgI/mL) supplied by Jiangsu Hengrui Medicine Co., Ltd. The second scan utilized the contrasting agent not used in the first, with each administration totaling 85 mL at a flow rate of 3.0 mL/s. Subsequently, 30 mL of a saline solution was injected at the same rate. The contrast agent injection protocol employed contrast agent tracking and automatic triggering scan technology, with a specific focus on the abdominal aorta as the region of interest. Monitoring of contrast agent arrival occurred simultaneously with injection of the contrast agent, and arterial phase imaging was automatically initiated 10 s after the CT value in the region of interest reached 100 Hounsfield units. Following a 25–30 s interval after the arterial phase, portal vein phase CT scanning commenced. Delayed phase imaging was conducted 70–90 s after the initiation of the portal vein phase. Thin-slice reconstruction was performed using a soft tissue window setting with a window width of 240 Hounsfield units, a window level of 50 Hounsfield units, a slice thickness of 2 mm, and a 2 mm interslice gap. To maintain consistency in scanning conditions, all procedures were performed by the same team of radiologic technologists, adhering strictly to the same scanning protocol.

### 2.3 Quantitative analysis

To ensure consistency in the selection of ROIs and to maximize the reduction of variability, this study adopted standardized protocols. Two radiologists, each with over 5 years of professional experience, independently delineated ROIs using the picture archiving and communication system (PACS) and assessed the quality of portal venous phase images, blinded to the study hypotheses. ROI selection deliberately avoided areas with artifacts or major vessels, with particular attention to placing ROIs in regions of homogeneous tissue enhancement. Before the commencement of the study, the radiologists participated in calibration exercises to ensure consistent ROI selection. A third radiologist reviewed a portion of the ROIs and performed an inter-rater reliability analysis on the data from the first two radiologists. Any discrepancies were resolved through consensus.

**Figure 1:**
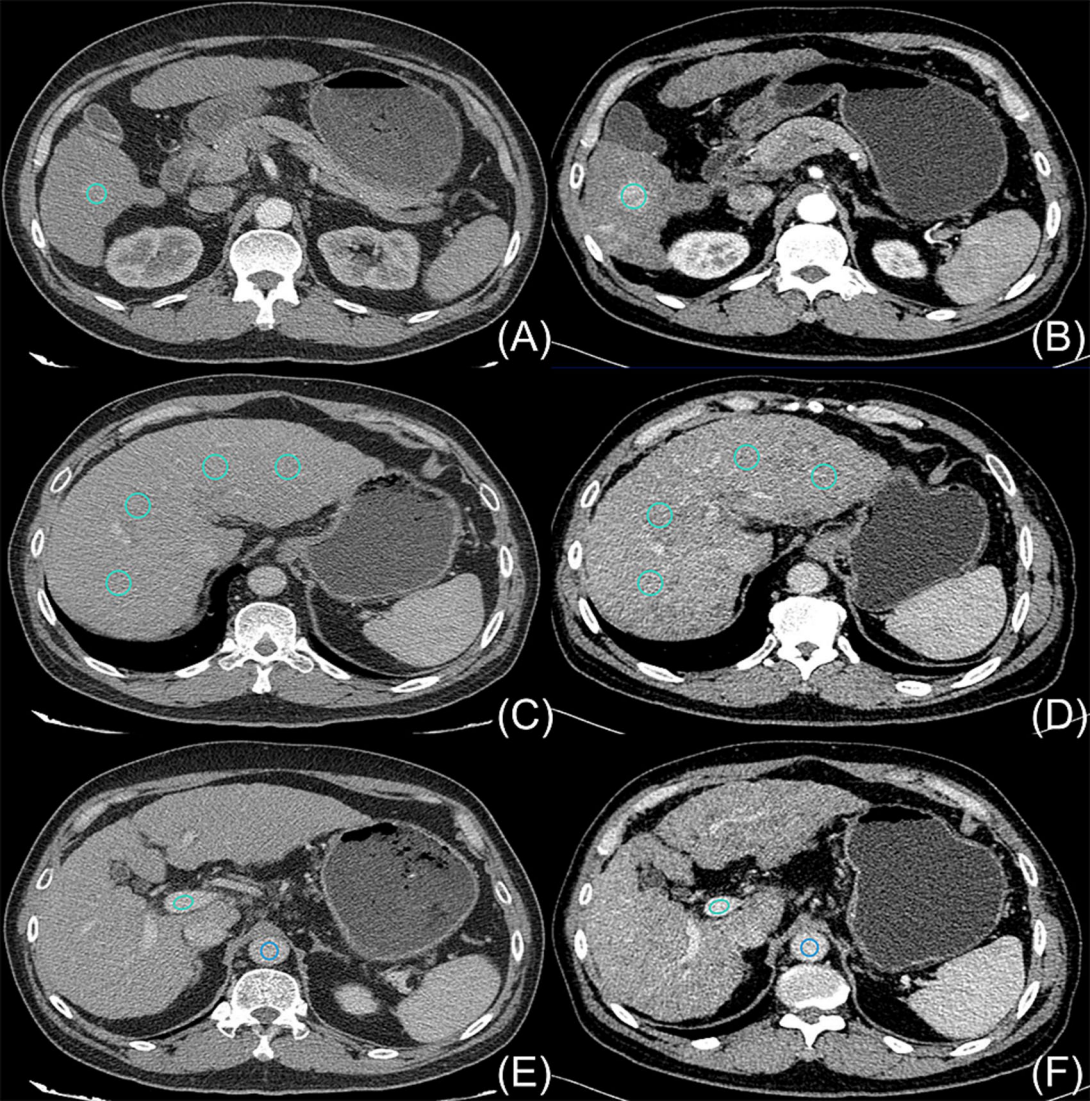
On 24 August 2018, a 54-year-old man with hepatocellular carcinoma underwent initial upper abdominal enhanced CT imaging in which a contrast agent concentration of 300 mg I/mL was used (A,C,E); this patient was subsequently examined on 6 November 2018 using an increased contrast agent concentration of 350 mg I/mL (B,D,F). The light blue circles indicate the ROIs outlining the hepatocellular carcinoma parenchyma (A,B), the liver parenchyma (C,D), and the portal vein (E,F). The dark blue circles in (E,F) indicate the ROIs outlining the aorta.

The radiologists manually measured the CT values (in Hounsfield units, HU) for the arterial, venous, and delayed phases of the liver cancer parenchyma imaging on cross-sections with a reconstructed layer thickness of 2 mm. To obtain the mean CT values of the HCC, liver parenchyma, abdominal aorta, and portal vein during different phases, circular or ovoid regions of interest (ROIs) measuring 100 ± 10 mm^2^ were placed at the level of the portal vein. The average CT values obtained by both radiologists were then calculated to generate the final results. The ROIs are outlined schematically in Figure 1.

The details of the measurement procedure are as follows:

1. To ensure anatomical uniformity, anatomical references such as the branches of the hepatic artery, the portal vein, or the hepatic vein, as well as the shapes of lesions caused by the hepatocellular carcinoma, along with the presence of additional structures such as the spleen, pancreas, ribs, and vertebrae, were considered for each patient. This approach was adopted to maintain consistency in the positioning and dimensions of both the initial and subsequent ROIs.
2. Four ROIs were delineated on the hepatic parenchyma; they were located in the right anterior-posterior and left medial-lateral regions of the right and left liver lobes. Particular care was taken to select areas that exhibited consistent and homogeneous enhancement and that maintained normal parenchymal characteristics. Any regions that displayed alterations in focal density, the presence of large blood vessels, or noticeable artifacts were deliberately excluded from consideration, and the resulting average values were calculated.
3. During the process of delimiting the hepatocellular carcinoma tissue, meticulous attention was given to excluding the incorporation of liquefied necrotic regions and guaranteeing the consistency of outlines in terms of both area and location.
4. In relation to the abdominal aorta, singular ROIs that encapsulated 90% or more of the cross-sectional area of each aorta were established. Particular emphasis was placed on circumventing the presence of calcifications and soft plaques within the aortic wall.
5. The portal vein was chosen as the reference point for measurement. To ensure consistency, all the measurements were repeated three times at three consecutive levels, and the mean of the three measurements was calculated.

### 2.4 Visual analysis

This study employed a 5-point scale to evaluate image quality, where 1 denotes poor quality and 5 signifies excellent quality. These scores were subsequently converted into categories for statistical analysis. The rating criteria are as follows:

1. 1 Point: Poor vessel enhancement, blurred borders, or severe artifacts hindering diagnosis.
2. 2 Points: Blurred vessel borders, still allowing the identification of the portal vein trunk and the assessment of obvious filling defects in portal vein branches of grades 1 and 2 but not in those of lower grades.
3. 3 Points: Clear visualization of the main portal vein trunk, enabling the evaluation of filling defects in grade 4 or higher branches.
4. 4 Points: Evident enhancement of blood vessels at all portal vein levels, albeit with slightly rough vessel borders.
5. 5 Points: Highly pronounced vessel enhancement with clear borders (9).

Two radiologists independently assessed the image quality and calculated the average score. A third radiologist reviewed a portion of the images and conducted a consistency analysis of the ratings provided by the initial two physicians.

### 2.5 Statistical analysis

Assessment of normality of the CT values for hepatocellular carcinoma parenchyma, liver parenchyma, aorta, and portal vein after enhancement in three stages was conducted using the Shapiro–Wilk test. A paired-samples *t*-test was applied to compare the CT values between the high and low iodine contrast concentration groups across different scan stages (hepatocellular carcinoma parenchyma, hepatic parenchyma, abdominal aorta, and portal vein).

Two physicians conducted a kappa consistency test to assess the subjective image quality. The hierarchical information obtained from the subjective image quality scores was then compared within matched groups via a paired-sample Wilcoxon signed rank-sum test. All statistical analyses were performed using IBM SPSS Statistics 24, with a significance level set at *p* < 0.05.

## 3 Results

The present study included 29 patients who were longitudinally divided into two groups on the basis of the iodine concentration of the contrast agent administered: a low-concentration group and a high-concentration group. The patient’s demographic and clinical characteristics are listed in Table 1.

**Figure 2:**
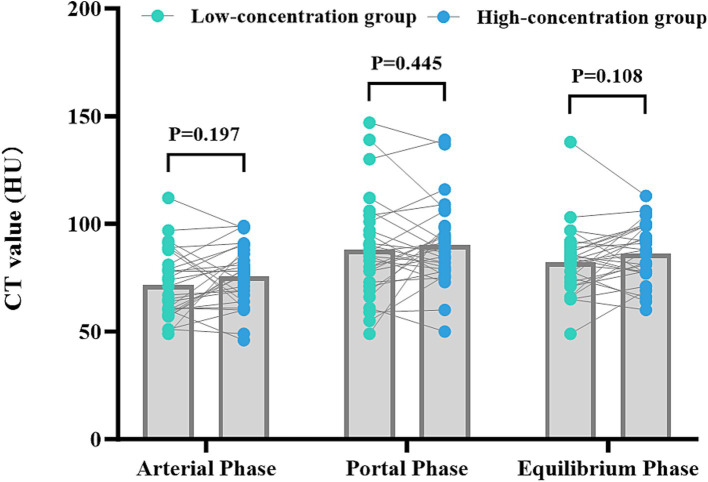
Comparison of the differences in CT values for parenchymal enhancement of hepatocellular carcinoma lesions between the two groups of hepatocellular carcinoma patients. The horizontal coordinates represent the scanning stage (arterial, portal venous, or delayed), and the vertical coordinates represent the mean CT values (HUs). There were no statistically significant differences in the CT values for parenchymal enhancement of hepatocellular carcinoma lesions between the two groups at any of the three scanning stages (*p* > 0.05).

**Figure 3:**
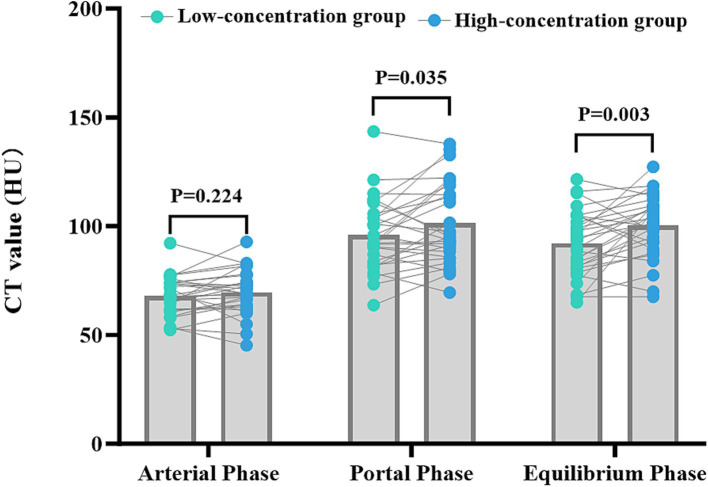
Comparison of the differences in liver parenchymal enhancement CT values between the two groups of patients. The horizontal coordinates represent the scanning stages (arterial, portal venous, and delayed), and the vertical coordinates represent the mean CT values (HUs). There was no statistically significant difference (*p* > 0.05) between the two groups of patients in the liver parenchymal enhancement CT values in the arterial phase. However, there were statistically significant differences (*p* < 0.05) between the two groups of patients in the liver parenchymal enhancement CT values in the portal venous and delayed phases, with patients in the high-concentration group having higher CT values.

**Figure 4:**
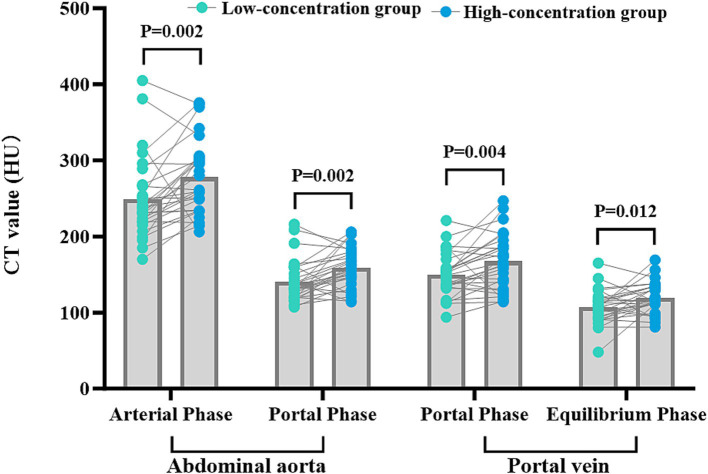
Comparison of CT values for abdominal aortic and portal vein enhancement in the two groups. The horizontal coordinates represent the scanning phases (arterial, portal, and delayed), and the vertical coordinates represent the mean CT values (HU). The CT values for abdominal aortic and portal vein enhancement in the arterial, portal venous, and delayed phases were significantly different in the two groups (*p* < 0.05), with higher CT values in the HU group.

The disparities in the CT values measured during the arterial phase, the portal venous phase, and the delayed phase in the high-and low-concentration groups of hepatocellular carcinoma patients were not statistically significant (*p* > 0.05) (Figure 2). The differences between the two groups in the CT values measured during the arterial phase of hepatic parenchymal enhancement were not statistically significant (p > 0.05) (Figure 3). However, significant differences between the two groups in the CT values during the portal venous phase and the delayed phase of hepatic parenchymal enhancement were observed (*p* < 0.05), and statistically significant differences in the CT values measured during the arterial phase, the portal venous phase, and the delayed phase of the aorta and portal vein were evident in both groups (*p* < 0.05) (Figure 4).

**Figure 5:**
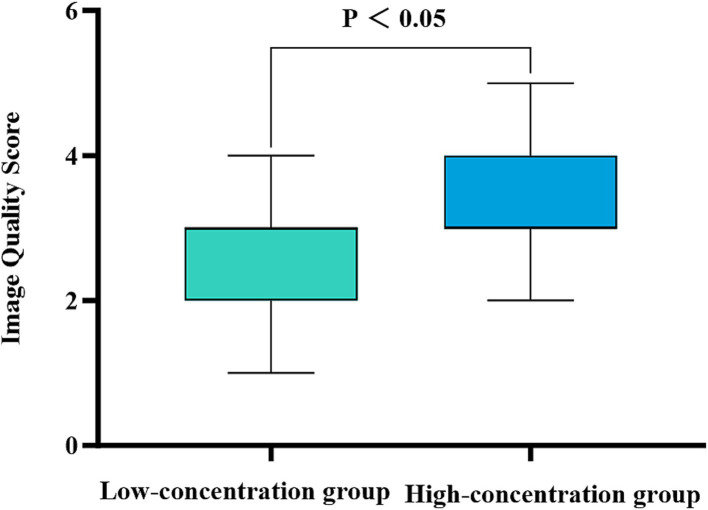
Comparison of the subjective image quality scores for the two groups of patients. The horizontal coordinate represents the scanning phase (arterial, portal venous, and delayed), and the vertical coordinate represents the average image quality score. The patients in the high-concentration group had significantly higher image quality scores than those in the low-concentration group (*p* < 0.001).

There was a substantial level of concordance between the two physicians in the subjective evaluation of image quality in the two groups as indicated by a high level of agreement (Kappa = 0.625, *p* < 0.001). The images obtained from patients in the low-concentration group were assigned image quality scores of 2.44 ± 0.12, whereas those obtained from patients in the high-concentration group achieved scores of 3.30 ± 0.67. In particular, the high-concentration group presented significantly higher scores than did the low-concentration group (*Z* = 3.819, *p* < 0.001), emphasizing the statistically significant difference (Figure 5).

## 4 Discussion

In this study, the effects of the concentration of iodine contrast agent used on the quality of CT images obtained from liver cancer patients were evaluated using an intraindividual comparison design. The results indicate that using a high concentration of contrast agent significantly improves the CT image values for the liver parenchyma, the abdominal aorta, and the portal vein as well as the image quality scores. This provides an important reference for the selection of the appropriate contrast concentration to be used in clinics. The results of this study thus support the use of high-concentration contrast agents in CT-enhanced scanning of patients with hepatocellular carcinoma. High-concentration contrast agents can provide better vascular enhancement and improve the contrast between the tumor and the surrounding tissues, thus improving image quality and contributing to the early detection and diagnosis of tumors. The use of high-concentration contrast can also enhance the visualization of the vascular structure of the abdominal aorta and portal vein, and this can help in vascular reconstruction and assessment, providing more accurate information for clinical treatment planning.

Several previous studies have supported that using high concentrations of contrast can improve the quality of CT images in patients with hepatocellular carcinoma (9–14). Jo et al. (11) used different concentrations of iodine contrast in two scans of patients with chronic liver disease and showed that the use of a high-concentration iodine contrast agent (Iomephenol 400) significantly improved CT values of the aorta, portal vein, and hepatic parenchyma in both the portal and delayed phases, thereby improving the image quality of each phase. This observation aligns with the findings of our study. Nevertheless, the study was constrained by an extended interval of approximately 271 days between the initial and follow-up CT scans. Despite the exclusion of patients with a weight change exceeding 5% during that period, conditions such as hepatomegaly and diffuse liver disease may have altered hepatic parenchymal perfusion, consequently impacting the contrast-enhanced features in the liver. In contrast, our study exclusively enrolled patients whose initial and follow-up CT scans were collected within a three-month interval. Furthermore, the introduction of a subjective image quality score further validates and demonstrates the robustness of our study results. In a circulation phantom study, Mihl et al. (15) observed that intravascular attenuation remained relatively stable when different contrast agents with varying iodine concentrations were administered at constant IDRs. It is possible to achieve target attenuation levels with contrast agent concentrations as low as 240 mg I/mL while keeping the IDR constant. However, this necessitates a higher injection rate, which may increase patient discomfort and the risk of extravasation. Conversely, it has been suggested that lower iodine contrast concentrations may be more evenly distributed within vessels because of their lower viscosity, potentially leading to a more consistent contrast distribution. This, in turn, could improve the visualization of smaller vessel segments (16). The advantage of a high IDR is partially offset by the slower and less uniform mixing of the contrast agent with the blood in the vessel, because of its higher viscosity and injection pressure. Additionally, individual patient factors such as heart rate, cardiac output, and body mass index add further complexity to the relationship between contrast concentration and vascular enhancement (17, 18). Nevertheless, the findings of our present study indicate that high-concentration contrast agents yield superior enhancement values compared to their low-concentration counterparts. The intrasubject controlled design adopted in our study offers greater statistical efficiency and clinical relevance over traditional independent sample designs, thus lending stronger support to our conclusions.

In specific clinical contexts, the use of a low concentration (300 mg I/L) of contrast material is advantageous for procedures such as CT screening and the diagnosis of hepatocellular carcinoma. Conversely, preoperative CT angiography requires a relatively high concentration of iodine contrast agent (350–370 mg I/L) to meet the standards of image quality. Although, generally, enhanced CT arterial phase images allow for arterial vasculature reconstruction, their quality is inferior to that achieved through CTA. It is worth noting that individuals diagnosed with hepatocellular carcinoma typically undergo a minimum of two CT-enhanced examinations throughout their diagnosis and treatment. This not only increases the financial burden but also raises concerns related to radiation exposure and potential physical harm to the patient.

The utilization of high-concentration contrast agents holds promise for simultaneously acquiring dual imaging modalities, streamlining examination procedures, and alleviating patient burden. The bolus volume of the high-concentration iodinated contrast agent was smaller than that of the low-concentration iodinated contrast agent (19). Administration of a high-concentration contrast agent at a fixed injection rate leads to rapid iodine delivery per unit time, resulting in earlier and greater peak arterial enhancement, albeit for a shorter period. However, hepatic enhancement remains unaffected (20). Under the premise of maintaining iodine flow rates, low-concentration iodinated contrast can also achieve comparable vascular imaging effects (21). However, this necessitates higher injection rates, and this may induce contrast extravasation, particularly in liver cancer patients, given their typically advanced age and the need for prolonged vascular access with associated drug stimulation, rendering administration of contrast at a high flow rate contrast administration impractical. Using a high-concentration iodinated contrast agent is an alternative approach that employs high injection rates to increase iodine delivery rates (16). Hence, increasing the contrast agent concentration is an optimal choice for liver cancer diagnosis and preoperative vascular reconstruction in a single examination setting.

In most recent assessments of the effectiveness of high-versus low-concentration imaging, the researchers have employed statistical methods to mitigate the effects of variations in fundamental patient characteristics. However, numerous patient-related factors, including body mass index, cardiac output, age, sex, venous access, and the presence of cirrhosis, portal hypertension, and other pathologies impacting organ enhancement, influence contrast imaging efficacy. The crossover design used in the current study was intended to mitigate the effects of several confounding variables, such as body weight, cardiac output, and the type and severity of cirrhosis, by utilizing each patient as their own control. However, our study has certain limitations. Although the time interval between the two consecutive CT examinations was restricted to 3 months and the order in which each contrast agent was used was randomized to reduce potential bias, we acknowledged that changes in tumor characteristics during this period could introduce variability. Factors such as tumor growth, the emergence of new lesions, or alterations in tumor vascularity might potentially influence the contrast enhancement patterns observed on CT scans. Moreover, this study focused solely on comparing 300 mg I/mL iohexol and 350 mg I/mL ioversol. Future research could consider including a wider variety of iodine contrast agents for comparative analysis. Additionally, as a single-center study with a relatively small sample size, the generalizability of the results may be limited. Therefore, future studies should increase the sample size, conduct large-scale, multicenter prospective studies, and perform stratified analyses based on disease stage, the presence or absence of cirrhosis, and other significant patient characteristics. This approach would further explore the differences in contrast agent concentration response among various patient populations, thereby providing more robust scientific evidence for clinical practice.

## 5 Conclusion

Our research demonstrated that the use of a high-concentration iodine contrast agent in routine CT enhancement scans of the upper abdomen effectively enhances the clarity of images of the liver parenchyma, the abdominal aorta, and the portal vein in patients with hepatocellular carcinoma. This enhanced imaging technique facilitates the reconstruction of hepatocellular carcinoma-associated blood vessels in patients in advanced stages of the disease, potentially limiting the need for supplementary CT examinations.

## Data Availability

The data analyzed in this study is subject to the following licenses/restrictions: the dataset used in support of the findings of this study are available from the corresponding author at email address upon request. Requests to access these datasets should be directed to PP, doublep@126.com.
